# Clean Water Should Be Recognized as a Human Right

**DOI:** 10.1371/journal.pmed.1000102

**Published:** 2009-06-30

**Authors:** The PLoS Medicine Editors

At the March 2009 United Nations (UN) meetings coinciding with the World Water Forum, Canada, Russia, and the United States refused to support a declaration that would recognize water as a basic human right. The special resolution proposed by Germany and Spain, and endorsed by the President of the UN General Assembly, was instead rejected in favor of further examination of issues of access to safe drinking water and sanitation [1].

Opposition to this declaration runs counter to considerable evidence that access to clean water, which is essential for health, is under threat. According to the World Health Organization, 1.2 billion people worldwide do not have access to clean drinking water, and a further 2.6 billion lack adequate sanitation services. These numbers are expected to rise. The UN has estimated that 2.8 billion people in 48 countries will be living in conditions of water stress or scarcity by 2025 [2].

Access to water should be framed as a human right for at least three reasons. First, ensuring access to clean water could substantially reduce the global burden of disease. Millions of people are affected each year by a range of water-borne diseases including cholera, hepatitis A, typhoid, and arsenic poisoning [3]. Diarrhea—a result of unsafe water and inadequate sanitation—is responsible for 1.8 million potentially preventable deaths per year, mostly among children under the age of five. Lack of water also results in poor hygiene: 6 million people worldwide are blind because of trachoma, the transmission of which can be dramatically reduced by simple hand washing [3]. The World Health Organization recently predicted that better access to safe drinking water and improvements in sanitation and hygiene could prevent 9.1% of the total burden of disease worldwide, or 6.3% of all deaths [4].

Second, the privatization of water—which exploits the view that water is a commodity rather than a public good—does not result in equitable access. For decades the World Bank, the World Trade Organization, and regional development banks have promoted private sector responsibility for water delivery [5]. This has led to the extensive privatization of water supply systems, especially in the developing world. The private model of water delivery now entails a US$400–US$500 billion global water industry that is dominated by three multinational companies who have, according to critics, neither proved their ability to provide sufficient or affordable water sources, nor effectively served the poor who suffer most from a lack of clean water [5],[6].

As Maude Barlow, senior advisor on water issues to the president of the General Assembly of the UN, has argued, “high water rates, cut-offs to the poor, reduced services, broken promises and pollution have been the legacy of privatization” [7]. And it's not just that delivery is in the hands of corporations; political control has shifted too: “The fact that water is not an acknowledged human right has allowed decision-making over water policy to shift from the UN and governments toward institutions and organizations that favour the private water companies and the commodification of water,” she says.

Governments who have experimented with national privatization schemes, such as those in Bolivia, Ghana, Peru, and Trinidad and Tobago, have faced fervent protests from citizens opposed to the privatization of their water supply systems [8]. Documented failures across the United States, Africa, Indonesia, and Latin America are contained in two recent collections of case studies by Food & Water Watch, a US-based nonprofit consumer organization [6],[9].

But even those most critical of private sector involvement in water admit that there is a potential role for corporations, perhaps limited to delivering water or supplying infrastructure, under a human rights framework that views water as a public good. Under such a model, governments must maintain their responsibilities to ensure sufficient, safe, affordable, and accessible water [10].

Third, the world is changing in ways that will both exacerbate water scarcity and threaten the quality of the current water supply. The problems of climate change, population growth, agricultural development, and industrial pollution are increasing and put enormous pressure on existing water sources. No nation, rich or poor, appears to be immune from a water crisis. The US is facing the greatest water shortages of its history. In 2003 its Government Accountability Office projected that at least 36 states would face water shortages because of a combination of rising temperatures, drought, population growth, urban sprawl, waste, and excess [11]. In Australia, severe drought in 2007 caused such dangerous water shortages in the Murray-Darling river basin, which contains 40% of the nation's farms and provides the bulk of its food supply, that the entire river system has now come under federal management requiring a AU$10 billion reinvestment [12]. In the developing world, as Professor Jonna Mazet, director of the wildlife health center at the University of California, Davis emphasizes, water scarcity has implications for emergent infectious disease “because when people and animals use the same water sources for drinking, bathing, and defecating, we can get serious contamination of drinking water and an increased risk of zoonotic disease” (personal communication).

Like many health and environmental problems, water scarcity will hit the poor first. Less water for poor families and their agriculture will exacerbate poverty and malnutrition; longer distances to fetch water will increasingly take people, mostly women, away from other daily tasks and schooling; and traveling for water will pose greater risks to the safety of both women and children in conflict areas. That most developing nations will lack the proper resources and infrastructure to deliver clean water and monitor water quality limits their ability to respond to the water crisis. A human rights approach to water recognizes the potential for inequity and ensures that the most vulnerable are not ignored [10].

Notwithstanding the differences in the causes and effects of water shortages across the world, establishing access to water as a human right affirms the need for global collective action. The goals of the first international “decade for water” were not met, and the prospects of the Millennium Development Goal (number seven) to “halve the proportion of people without secure access to safe drinking water and adequate sanitation by 2015” appear dire. Critics have called inadequate access to water and sanitation a “silent emergency” that has yet to command sufficient attention from the international community or from health professionals [13],[14]. Clearly we need a more radical approach.

A human rights framework offers what the water situation needs—international recognition from which concerted action and targeted funding could flow; guaranteed standards against which the protected legal right to water could be monitored; and accountability mechanisms that could empower communities to advocate and lobby their governments to ensure that water is safe, affordable, and accessible to everyone.
